# Supplementary material for: MetaRibo-Seq measures translation in microbiomes
Source: Nat Commun. 2020 Jun 29;11:3268. doi: 10.1038/s41467-020-17081-z (PMC7324362; doi:10.1038/s41467-020-17081-z)
Supplement: Supplementary file 10 — Supplementary Data 7 [file 41467_2020_17081_MOESM10_ESM.zip › File2/Confidence_High_Taxonomy/263517_out.krona.html]

Javascript must be enabled to view this page.

members
magnitude
magnitudeUnassigned
count
unassigned
taxon
rank

263517\_out

13

13
superkingdom
2

phylum
976
13

class
200643
13

171549
order
13

13
family
815

13
genus
816

10

SRS046068\_contig\_number\_238SRS049900\_contig\_number\_15043SRS050026\_contig\_number\_11703SRS063928\_contig\_number\_20SRS064645\_contig\_number\_932SRS075773\_contig\_number\_24135SRS078665\_contig\_number\_10074SRS098073\_contig\_number\_16668SRS100021\_contig\_number\_7882SRS148091\_contig\_number\_2230
820
species

3

SRS016095\_contig\_number\_357SRS062701\_contig\_number\_6391SRS1055038\_contig\_number\_566
291645
species
